# Stereotactic Body Radiation Therapy for Hepatocellular Carcinoma: Prognostic Factors of Local Control, Overall Survival, and Toxicity

**DOI:** 10.1371/journal.pone.0077472

**Published:** 2013-10-11

**Authors:** Jean-Emmanuel Bibault, Sylvain Dewas, Claire Vautravers-Dewas, Antoine Hollebecque, Hajer Jarraya, Thomas Lacornerie, Eric Lartigau, Xavier Mirabel

**Affiliations:** 1 Academic Radiation Oncology Department & University Lille II, CLCC Oscar Lambret, Lille, France; 2 Department of Medicine, Institut Gustave Roussy, University Paris 11, Villejuif, France; 3 Department of Radiology, CLCC Oscar Lambret, Lille, France; University of Modena & Reggio Emilia, Italy

## Abstract

**Purpose:**

Stereotactic body radiation therapy (SBRT) for hepatocellular carcinoma (HCC) has been evaluated in several recent studies. The CyberKnife^®^ is an SBRT system that allows for real-time tracking of the tumor. The purpose of this study was to evaluate the prognostic factors for local control and overall survival following this treatment.

**Patients and Methods:**

75 patients with 96 liver-confined HCC were treated with SBRT at the Oscar Lambret Comprehensive Cancer Center. Fiducials were implanted in the liver before treatment and were used as markers to track the lesion’s movement. Treatment response was scored according to RECIST v1.1. Local control and overall survival were calculated using the Kaplan and Meier method. A stepwise multivariate analysis (Cox regression) of prognostic factors was performed for local control and overall survival.

**Results:**

There were 67 patients with Child-Turcotte-Pugh (CTP) Class A and eight patients with CTP Class B. Treatment was administered in three sessions. A total dose of 40–45 Gy to the 80% isodose line was delivered. The median follow-up was 10 months (range, 3–49 months). The local control rate was 89.8% at 1 and 2 years. Overall survival was 78.5% and 50.4% at 1 and 2 years, respectively. Toxicity mainly consisted of grade 1 and grade 2 events. Higher alpha-fetoprotein (aFP) levels were associated with less favorable local control (HR=1.001; 95% CI [1.000, 1.002]; p=0.0063). A higher dose was associated with better local control (HR=0.866; 95% CI [0.753, 0.996]; p=0.0441). A Child-Pugh score higher than 5 was associated with worse overall survival (HR= 3.413; 95% CI [1.235, 9.435]; p=0.018).

**Conclusion:**

SBRT affords good local tumor control and higher overall survival rates than other historical controls (best supportive care or sorafenib). High aFP levels were associated with lesser local control, but a higher treatment dose improved local control.

## Introduction

Hepatocellular carcinoma (HCC) has become one of the leading causes of death in cirrhotic patients [1]. Tumour stage, liver function and patient general condition are the main prognostic factors in HCC. Among the available classifications, the Barcelona Clinic Liver Cancer (BCLC) classification is endorsed by both US and European associations for the study of liver diseases and oncology [2]. Surgery is the standard of care for these patients, but only 10–30% of patients are eligible for this treatment. When surgery is not possible, therapeutic options include percutaneous alcohol injection, radiofrequency ablation (RFA) [3] and transarterial chemoembolization (TACE) [4]. When these options are not feasible, recommendations include best supportive care, sorafenib [5] or radiotherapy [6], performed alone or in combination [7].

Three local modalities are actually recognized as curative treatment options for patients with early-stage hepatocellular carcinoma: liver transplant, surgical resection, and percutanuous radiofrequency ablation. Palliative treatments include sorafenib, radiotherapy and TACE [8]. Conformal radiotherapy and stereotactic body radiotherapy (SBRT) are not included as curative options in the European Association for the Study of the Liver practical guidelines [9] and are consequently rarely discussed during tumor boards. 

Stereotactic body radiotherapy (SBRT) delivers high radiation doses with precision and has been used to treat patients without other therapeutic options in clinical situations as diverse as inoperable recurrent head and neck squamous-cell carcinoma [10] or pelvic recurrences in a previously irradiated area [11]. Liver SBRT, pioneered almost 20 years ago by Blomgren et al. [12], can be used to treat secondary [13] or primary tumors and is an option for local treatment of HCC patients ineligible for surgery, TACE, chemotherapy, or RFA. This treatment technique has shown enticing rates of local control and low toxicity [14]. Ultimately, SBRT could be an alternative to existing local treatments. CyberKnife^®^ (Accuray Incorporated, Sunnyvale, California, U.S.A.) is a robotic image-guided system that delivers hypofractionated SBRT with intrafraction motion detection and correction. Other possible techniques include Novalis Tx^®^ (Brainlab AG, Feldkirchen, Germany) and TrueBeam^®^ (Varian, Palo Alto, California, U.S.A.).

We already published results in patients treated for primary and secondary liver lesions [15]. This study reports the results of SBRT in an updated and expanded population of patients treated for hepatocellular carcinoma only. The primary objective was to determine the prognostic factors of tumor local control and overall survival for these patients, in order to better distinguish patients who are most likely to benefit from this treatment option from those that are not.

## Materials and Methods

Seventy-five HCC patients (12 women and 63 men) were treated with SBRT from July 2007 to November 2011. The patients included in the study had HCC, an Eastern Cooperative Oncology Group (ECOG) performance score of less or equal to 2, pre-treatment Child-Turcotte-Pugh (CTP) (Table 1) scores A5–B8, and ineligibility for surgical resection, TACE, RFA, or sorafenib. Main reasons for ineligibility were inoperable patients and size or location of the tumor incompatible with RFA or TACE procedures. Diagnosis was established based on biopsy or according to the Barcelona criteria [16]. Patients could have no more than three lesions in order to qualify. Any previous treatments could include TACE, chemotherapy, surgery, or RFA. Patients did not have any distant metastases. Median follow-up for surviving patients was 10.0 months (range, 3–49 months).

 Cases were discussed in a multi-disciplinary HCC board consisting of a hepatologist, a hepatic surgeon, a radiation oncologist, a medical oncologist, and a radiologist. This retrospective, single-institution, cross-sectional study was approved by our Institutional Committee on Human Research.

### Treatment Planning and Delivery

All patients were treated with CyberKnife. Real-time tracking of tumor movements was performed with MultiPlan^®^ (Accuray) treatment planning software and Synchrony^®^ (Accuray) respiratory tracking system. Gold seeds measuring 0.88 mm in diameter by 5 mm in length (Ab Medica, Milan, Italy) were implanted around each lesion. Treatment planning CTs were performed at least 7 days after fiducial placement. Patients were immobilized either in a vacuum mattress or a self-expanding foam mattress in the treatment position (supine). A spiral CT scan without contrast and a three-phase scan with contrast (arterial, portal, and late phases) were acquired for planning. Slice thickness was 1 mm. 4D-CT was not used. The gross tumor volume (GTV) was contoured on the contrast-enhancing disease visible on the partial exhale contrast-enhanced CT scan. Patients were asked to exhale and then hold their breath thereby eliminating the nooed for a gated scan. Tumor tracking was performed during treatment using the inserted fiducials. The clinical target volume (CTV) was defined as the GTV with a geometrical 10-mm margin in all directions within the liver (institutional standard). A 1.5 mm margin was applied to the CTV to obtain the planning target volume (PTV). A total dose of 24–45 Gy in three fractions of 8-15 Gy each was prescribed to the 80% isodose line (95% PTV coverage) and delivered to the PTV over 10–12 days. The dose was adjusted as necessary in order to remain within the dose constraints of the normal tissues and surrounding organs at risk. Dose constraints are shown in table 1.

**Table 1:** Dose constraints for critical structures (treatment in three fractions).

Spinal cord	V16 < 1.2 cm3
	V18 < 0,25 cm3
	max 22 Gy
Lungs (Right + Left)	V5 < 50 %
	V10 < 30 %
	(Vtotal - V11) > 1500 cm3
Heart	V24 < 15 cm3
	max 30 Gy
Vessels	V39 < 10 cm3
	max 45 Gy
Esophagus	V15 < 10 cm3
	V21 < 5 cm3
	V25 < 0,5 cm^2^
Remaining healthy liver	V15 < 50 %
	V21 < 33%
	(Vtotal-V17) > 700 cm3
Stomach	V19 < 10 cm3
	V21 < 5 cm3
	V25 < 0,5 cm3
Duodenum	V15 < 5 cm3
	V24 < 0,5 cm3
Small intestines	V16 < 5 cm3
	V27 < 0,5 cm3
Colon	V20 < 20 cm3
	V30 < 1 cm3
Kidney	V10 < 50 %
	(Vtotal - V15) > 200 cm3

### Patient follow-up

Each patient had a clinical and biological evaluation 6 weeks after the completion of the treatment. Clinical, and radiological follow-up was performed every 3 months during the first 15 months following treatment and every 6 months thereafter. At each follow-up visit, a CT scan or MRI was obtained. All images were reviewed by a radiologist who classified responses as partial, complete, or progressive disease based on the modified Response Evaluation Criteria in Solid Tumors (mRECIST)[17]. Toxicity was evaluated according to the National Cancer Institute (NCI) Common Terminology Criteria for Adverse Events (CTCAE) v4.0[18]. 

### Statistics

The R statistical package version 2.15.0 (R Development Core Team, 2012) was used for the statistical analyses. Time to local failure was defined from the last treatment session. Rates were estimated using the Kaplan-Meier method. Differences among survival curves were compared using the log-rank test. The univariate analyses of local control were performed using the Cox regression model. A p value <0.05 was chosen as the significance threshold. A stepwise multivariate analysis with cox regression of prognostic factors was performed for local control and overall survival.

### Ethics

This study was approved by the internal ethic board of our institution (Clinical Trial Commission; ''Commission interne des études cliniques''). Our institutional review board waived the need for written informed consent from the participants.French laws (Data, data-collection and freedom law, January, 6th 1978)state that in case of single-centre, retrospective study based on already recorded and stored data, there is no need of specific written informed consent. 

All patients have been orally informed about the potential use of their collected data for future research.Agreement N1034071 was obtained from the "National Commission about Data-collection and Freedom’’ (‘‘Commission Nationale Informatique et Liberte´’’) for the conduct of this study.

## Results

### Patient and treatment characteristics

Median age was 70 years (range, 44–86 years). Cirrhosis was due to alcohol consumption for 57 patients (76%), to viral hepatitis for11 patients (14.9%), to hemochromatosis for 6 patients (8.1%) and to NASH for 1 patient (1%). Median alpha-fetoprotein (AFP) level was 18.8 ng/ml (range, 1–3091 ng/ml). A majority of the patients (44 patients, 60.3%) had CTP A cirrhosis. Half of the patients had already received treatment for HCC (51% of the treated targets), the majority with chemoembolization (22 targets, 23.7%). One patient (1,3%) had already received radiation therapy. Median tumor diameter was 37 mm (range, 30–145 mm). Median number of treated lesions per patient was 1. Patients’ characteristics are shown in Table 2 Median sum of GTVs was 130.7 cm^3^ (range, 34–713.7 cm^3^) and median hepatic volume was 1557 cm^3^ (range, 841–3432 cm^3^). In most cases, four fiducials were inserted near the tumor before the treatment (61.3% of cases). Mean fraction length was 103.9 minutes (range, 35.7–156 minutes), median number of beams per treatment was 148 (range, 29–254 beams), and median total dose was 45 Gy (range, 24–45 Gy) with a mean 15 Gy (range, 8–15 Gy) delivered per fraction. Treatment characteristics are presented in Table 3.

**Table 2:** Patient characteristics.

	**Total N (%) or Median (range)**
**Gender - n (%)**	
**Male**	63 (84)
**Female**	12 (16)
**Age - years [95% CI]**	70 [55-85]
**ECOG Performance Status - n (%)**	
**0**	49 (66.2)
**1**	21 (27)
**2**	5 (6.8)
**Cause of cirrhosis - n (%)**	
**Alcoholic**	57 (76)
**Viral**	11 (14.9)
**Hemochromatosis**	6 (8 .1)
**NASH**	1 (1)
**AFP - median in ng/ml [95% CI]**	18.8 (1 - 3091)
**Child-Pugh score - n (%)**	
**5**	44 (58.7%)
**6**	22 (29.3%)
**7**	6 (8%)
**8**	3 (4%)
**MELD score - n (%)**	
**1-5**	12 (16%)
**5-10**	37 (49%)
**10-15**	19 (25%)
**15-20**	7 (9.3%)
**OKUDA score - n (%)**	
**1**	65 (86.7%)
**2**	10 (13.3%)
**CLIP score - n (%)**	
**0**	49 (65.3%)
**1**	18 (24%)
**2**	6 (8%)
**3**	2 (2.7%)
**BCLC score - n (%)**	
**A1**	23 (30.7%)
**A2**	8 (10.7%)
**A3**	1 (1 .3%)
**A4**	15 (20%)
**B**	10 (13.3%)
**C**	18 (24%)
**Prior treatments - n (%)**	49 of 96 target lesions (51%)
**Radiofrequency**	8 (8.6%)
**Surgery**	5 (5.4%)
**Chemo-embolization**	22 (23.7%)
**Radiotherapy**	1 (1.1%)
**Chemotherapy**	13 (14%)
**Hepatic volume - median in cm^3^ [95% CI]**	1557 (841- 2273)
**Targets size - median tumor diameter in mm [95% CI]**	37 (30-44)
**Median number of treated lesion per patient**	1 (1-3)

**Table 3:** Treatment characteristics.

	**Total N (%) or Median (range)**
**Number of treated targets**	96
**0**	58 (60.4%)
**1**	32 (33.3%)
**2**	6 (6.3%)
**Number of fiducials inserted**	
**1**	1 (1%)
**2**	2 (2.6%)
**3**	11 (14.6%)
**4**	46 (61.3%)
**5**	13 (17.3%)
**6**	2 (2.6%)
**Sum of GTV volumes (cm^3^)**	130.7 (34--713.7)
**Session length (min)**	103.8 (35.7--156)
**Number of beams**	148 (29--254)
**Total dose (Gy)**	45 (24--45)
**Dose per fraction (Gy)**	15 (8--15)

### Tumor local control rate

**Figure 1:**
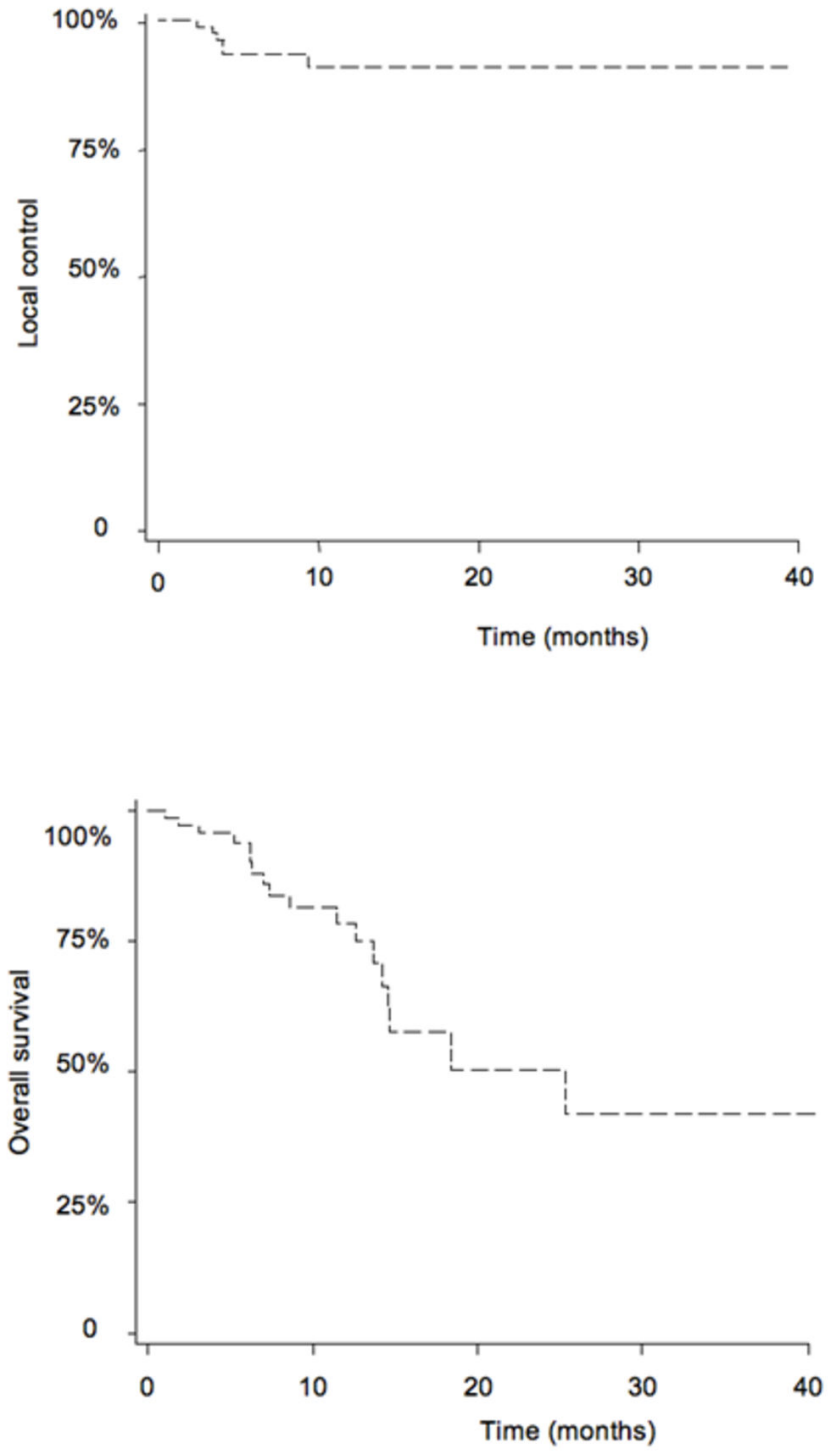
Kaplan Meier curves for local control and overall survival.

Ninety-six targets were treated and individually evaluated for 75 patients. The actuarial 1- and 2-year local control rate was 89.8%. Seven targets (7.3%) in six patients (8%) locally progressed after treatment. Eighteen hepatic recurrences (24%) at a distance from the target were observed. Six patients had distant metastasis after treatment. Kaplan-Meier curve for tumor local control is presented in Figure 1.

We performed a univariate analysis to identify prognostic factors affecting local control. Patients with a Cancer of the Liver Italian Program (CLIP) score higher than 2 were more likely to relapse (HR=10.127; CI95=2.182, 47.12; p=0.0031). A lower risk of relapse was observed when a higher radiation dose per fraction was administered (HR=0.6473; CI95=0.492, 0.851; p=0.0018) and a higher total dose was delivered (HR=0.8634; CI95=0.789, 0.945; p<0.0001). In multivariate analysis, a higher total dose remained significantly associated with a lower risk of relapse (HR=0.866; CI95=0.753, 0.996; p=0.0441). AFP was also associated with higher risk of local failure (HR=1.0011; CI95=1.001, 1.002; p<0.0001). Results are presented in Table 4. 

**Table 4:** Prognostic factors for local control (n=96 lesions).

**Factor**	**Hazard Ratio (95% CI)**	**p value**
*Univariate analysis*		
**AFP**	1.0011 (1.001,1.002)	<.0001
**Child-Pugh score**	0.6664 (0.061,7.303)	0.7397
**MELD score**	1.0345 (0.907,1.180)	0.6135
**OKUDA score**	1.6909 (0.178,16.018)	0.6471
**CLIP score = 1**	0.8346 (0.082,8.472)	0.8785
**CLIP score = 2**	10.1277 (2.182,47.012)	0.0031
**Previous treatment**	1.2377 (0.263,5.835)	0.7875
**Previous chemo-embolization**	1.3802 (0.237,8.049)	0.7202
**Previous chemotherapy**	2.6016 (0.398,16.999)	0.3181
**Number of treated lesions**	0.7643 (0.103,5.652)	0.7923
**Sum of GTV volumes**	1.0064 ( 1.004,1.009)	<0.0001
**Total dose**	0.8634 (0.789,0.945)	<0.0001
**Dose / fraction**	0.6473 (0.492,0.851)	0.0018
*Multivariate analysis*		
**AFP**	1.001 (1.000,1.002)	0.0063
**Total dose**	0.866 (0.753,0.996)	0.0441

### Overall survival rate

Disease-free survival (DFS) was 61.7% at 1 year and 31.8% at 2 years. A univariate analysis identified two factors associated with DFS. Targets already treated with chemoembolization were more likely to relapse (HR=2.9595; CI95=1.390, 6.302; p=0.0049). Higher doses per fraction were associated with better DFS (HR=0.7537; CI95=0.632, 0.899; p=0.0016). Overall survival at 1 and 2 years were 78.5% and 50.4%, respectively. The median overall survival was 15 months (95% CI). The Kaplan-Meier curve for overall survival is presented in Figure 1. A univariate analysis followed by a multivariate analysis of the factors associated with overall survival was performed. In univariate analysis, patients with a CTP score higher than 7 appeared to have shorter overall survival (HR=4.3309; CI95=1.128, 16.624; p=0.0327). In line with this, patients with more than two treated targets also had shorter overall survival (HR=6.2630; CI95=1.549, 25.327; p=0.0101). The multivariable analysis included the following factors: alcohol consumption, AFP initial measurements, CTP score (<=7 vs >7), CLIP score (<=1 vs >1), number of treated targets (=1 vs >1), sum of the size of the treated lesions, sum of the GTVs, sum of the PTVs and total dose, dose/fraction.CTP score higher than 7 remained a significant predictive factor associated with worse overall survival (HR=3.413; CI95=1.235, 9.435; p=0.0180). Detailed results are presented in table 5.

**Table 5:** Prognostic factors for overall survival (n=75 patients).

**Factor**	**Hazard Ratio (95% CI)**	**p value**
*Univariate analysis*		
**Age**	1.0044 (0.955,1.056)	0.8635
**Obesity**	2.3596 (0.957,5.819)	0.0623
**AFP**	0.9994 (0.998,1.000)	0.294
**Child-Pugh score <7 vs >7**	4.3309 (1.128, 16.624)	0.0327
**MELD score**	1.0388 (0.930,1.161)	0.5017
**OKUDA score**	2.3761 (0.708,7.971)	0.1611
**CLIP score = 1**	1.5858 (0.594,4.235)	0.5920
**CLIP score = 2**	0.5302 (0.052,5.397)	0.5920
**Previous treatment**	1.0045 (0.404,2.5)	0.9923
**Number of treated lesions**	0.9010 (0.247,3.282)	0.8744
*Multivariate analysis*		
**Child-Pugh score <7 vs >7**	3.413 (1.235,9,.435)	0.0180
**CLIP score = 2**	0.676 (0.088,5.208)	0.7066
**Number of treated lesions**	2.035 (0.661,6.263)	0.2157

### Toxicity from radiation exposure

Overall the treatments were well tolerated. The most common toxicities were hepatic pain (17.1%), nausea (17.1%), vomiting (15.8%), and asthenia (15.8%), with most patients having a grade 1 or grade 2 toxicity. No radiation-induced liver disease (RILD) was observed, but five patients (6.6%) had decompensated cirrhosis three months later. One of the first treated patients, presented with a grade 4 gastric ulcer following treatment, resulting in a digestive hemorrhage. For this patient, the treated target was in the proximity of the stomach. Following observation of this toxicity, the treatment plans were reviewed and strict dose constraints were defined for subsequent treatments (V15 Gy<3 cm^3^). Toxicities are summarized in table 6. A univariate analysis was performed to find prognostic factors for hepatic pain, hyperbilirubinemia, nausea, vomiting, asthenia, and decompensated cirrhosis. No factor was predictive for hepatic pain or hyperbilirubinemia. Treatment session duration was predictive of nausea (OR=1.029; p=0.0477), vomiting (OR=1.034; p=0.04). The aggregate sum of the Gross Tumor Volumes was also predictive of vomiting (OR=1.004; p=0.0236), i.e., patients with larger tumors had a more elevated risk of vomiting. Patients with larger healthy liver volumes were less likely to decompensate their cirrhoses (OR=0.998; p=0.006).

**Table 6:** Toxicities (n=75 patients).

**Toxicity**	**Total N (%)**
**Duodenal ulcer Grade 2**	3 (4%)
**Gastric ulcer Grade 4**	1 (1.3%)
**Hepatic pain**	13 (17.1%)
**Grade 1**	8 (10.6%)
**Grade 2**	2 (2.7%)
**Grade 3**	3 (4%)
**Nausea**	13 (17.1%)
**Grade 1**	9 (12%)
**Grade 2**	4 (5.3%)
**Vomiting**	12 (15.8%)
**Grade 1**	8 (10.6%)
**Grade 2**	4 (5.3%)
**Fatigue**	12 (15.8%)
**Grade 1**	3 (4%)
**Grade 2**	6 (8%)
**Grade 3**	3 (4%)
**Diarrhea**	0 (0%)
**Decompensated cirrhosis (ascites)**	5 (6.6%)

## Discussion

Stereotactic body radiotherapy (SBRT) is not included as a curative option in the European Association for the Study of the Liver practical guidelines [9]. Of note, concerns have been raised about the possibility of radiation-induced liver disease for patients treated with external-beam radiotherapy (EBRT), which encumbered the inclusion of EBRT in the therapeutic strategies for cirrhotic patients. This is despite almost 20 years have now passed since Blomgren et al. have pioneered SBRT for liver malignancies [12]. 

Since then, most studies published about SBRT for liver tumors have contained both liver metastases and hepatocellular carcinoma. Articles about SBRT for HCC alone are scarce. Most of these studies are retrospective, like ours, or with a very limited number of patients. We present here the largest study in terms of both patients (75) and the number of lesions (96) treated. 

In Blomgren’s articles, 42 tumors in 31 patients (eight with HCC) were treated. Dose per fraction ranged from 7.7 to 30 Gy (mean, 14.2 Gy). Treatments consisted of one to four fractions delivering a total mean dose of 8 to 66 Gy to the PTV, respectively. The local control rate in the study was 80%. Since then, SBRT for HCC has been explored as a local salvage treatment after incomplete transarterial TRACE [19], as a bridge to transplantation [20] or for recurrence treatment [21]. 

Four prospective studies have been published regarding patients with HCC treated with SBRT. In the study by Mendez-Romero et al [22], 25 patients with liver tumors, including eight patients with HCC, were treated. Two fractionation regimens were prescribed depending on the tumor diameter, i.e. , patients with tumors smaller than 4 cm received 37.5 Gy in three fractions, while patients with tumors larger than 4 cm had 25 Gy in five fractions. Patients who presented with local failure were all in this latter group. Due to the occurrence of these failures, the dose was increased to 30 Gy in three fractions for patients with tumors larger than 4 cm. One-year local control was 75% and 1-year survival rate 48%. One patient with CTP B cirrhosis experienced grade 5 RILD. Two years later, Tse et al published their own prospective study, a phase 1 trial with 41 patients among whom 31 had HCC [23]. Radiation dose was adapted according to predicted liver toxicity, based on a normal tissue complication model [24]. Median dose was 36 Gy (range, 24–54 Gy) in six fractions. Median tumor volume was 173 cm^3^ (range, 9–1913 cm^3^). No patients were diagnosed with RILD, although 26% had grade 3 liver enzymes elevation. One-year local control rate was 65% and median survival was 11.7 months. In 2010, Cardenes published a phase I trial of SBRT for HCC with 17 patients (25 lesions). No dose-limiting toxicity was reported following an escalated 48 Gy in three fractions introduced in one to two fractions per week to patients with Child-Turcotte-Pugh (CTP) A cirrhosis. In line with previous studies, patients with CTP B cirrhosis experienced higher toxicity: four out of the five CTP B patients had grade 3 or higher liver toxicity, with an overall survival less than 6 months. 

Three retrospective studies have been published with HCC-only patients treated with SBRT. Forty-two patients were treated with 30 to 39 Gy in three fractions in the Kwon et al study [25]. Median follow-up was 29 months. At 1 and 3 years, in-field-progression-free survival was 72% and 68%, respectively. Overall 1- and 3-year survival rates were 92.9% and 58.6%, respectively. Patients experienced grades 1–2 toxicity but one patient died with extrahepatic metastasis and radiation-induced hepatic failure. The same year, Seo et al published a study including 38 patients with HCC treated with 33 to 57 Gy in three to four fractions [26]. Local control rate and overall survival were 79% and 68%, respectively. Dose was identified as an independent prognostic factor for survival, i.e., patients treated with 42 Gy in three fractions had an 81% 2-year survival rate, while patients who received a lower dose had a 25% rate. It is, nevertheless, difficult to draw strong conclusions considering the limited number of patients in this study. Six patients experienced a decline in liver function and one patient had a grade 3 musculo-skeletal toxicity. More recently, Andolino et al have reported on 60 patients treated with SBRT for liver-confined HCC [27]. Like in the Mendez-Romero study, dose was adapted to the CTP score of the patients, i.e., the median number of fractions, dose per fraction, and total dose were three, 14 Gy, and 44 Gy, respectively, for those with class A cirrhosis and five, 8 Gy, and 40 Gy, respectively, for those with class B. The 2-year local control and overall survival were 90% and 67%, respectively. Regarding toxicity, 13% of the patients experienced an increase greater than one grade of hematologic and hepatologic dysfunction.

More recently, two studies were reported regarding patients with active HCC unsuitable for standard locoregional therapies treated with SBRT [28]. 102 patients were evaluable : local control at 1 year was 87% (95% CI, 78% to 93%). Like in our study, SBRT dose was associated with local control on univariate analysis. Median overall survival was 17.0 months (95% CI, 10.4 to 21.3 months). This study and our study’s overall survival rates compare favorably with best supportive care and even with sorafenib [29,30] the only other potentially available therapy for these patients.

In these seven studies, SBRT showed high local control rates in the range of 70–90% at 1 year. Our results, regarding local control is similar to what has already been reported in the previous studies, suggesting that SBRT is an effective treatment, even for those previously treated for HCC. We showed that total dose was a prognostic factor for local control in multivariate analysis, further supporting the importance of this factor for the success of the treatment. In the present study, the 2-year survival rate was 50.4%, which is slightly lower than that in the other studies. This could be attributed to the fact that only ineligible patients for surgery, or those with relapsed HCC after a failed prior treatment, were included, indicating that these patients had less favorable prognoses to begin with. This higher mortality cannot be attributed to treatment toxicity, since it was found to be very low. In view of the large proportion of multifocal tumors in our study population and the difficult tumor sites for operation, this slightly lower survival rate is not surprising.

CTP B is recognized as a prognostic factor for overall survival and our analysis confirmed that patients with a higher score had lower overall survival. This could also hint at the fact that patients with advanced cirrhosis are more likely to die from liver failure. The toxicity of SBRT consists mainly of liver dysfunction. Other toxicities (pain, fatigue, nausea, and vomiting) were not dose-limiting, which was also the case in our study. A particular attention should be paid to patients with CTP B score since they are more likely to experience liver failure after SBRT. Our study did not confirm that result, even though we did find that patients with larger healthy liver volumes were less like to decompensate their cirrhosis.

## Conclusion

SBRT is an effective and safe treatement. In a population of patients without other curative local treatment options, SBRT shows sustained local control, associated with survival rates higher than historical controls, with a low risk of serious toxicity. Further randomized trials are required to compare his efficacy and safety to RFA and TACE.

## Supporting Information

Table S1 **The Child-Pugh Scoring System.**
(DOC)
