# Supplementary material for: Stereotactic Body Radiation Therapy for Hepatocellular Carcinoma: Prognostic Factors of Local Control, Overall Survival, and Toxicity
Source: PLoS One. 2013 Oct 11;8(10):e77472. doi: 10.1371/journal.pone.0077472 (PMC3795696; doi:10.1371/journal.pone.0077472)
Supplement: Table S1 — The Child-Pugh Scoring System. (DOC) [file pone.0077472.s001.doc]

Table S1

| **Measure** | **1 point** | **2 points** | **3 points** |
| --- | --- | --- | --- |
| [Total bilirubin](http://en.wikipedia.org/wiki/Bilirubin), μmol/l (mg/dl) | <34 (<2) | 34-50 (2-3) | >50 (>3) |
| [Serum albumin](http://en.wikipedia.org/wiki/Serum_albumin), g/l | >35 | 28-35 | <28 |
| [PT INR](http://en.wikipedia.org/wiki/Prothrombin_time#International_normalized_ratio) | <1.7 | 1.71-2.30 | > 2.30 |
| [Ascites](http://en.wikipedia.org/wiki/Ascites) | None | Mild | Moderate to Severe |
| [Hepatic encephalopathy](http://en.wikipedia.org/wiki/Hepatic_encephalopathy) | None | Grade I-II (or suppressed with medication) | Grade III-IV (or refractory) |
